# Association of preconception cannabis use frequency with cannabis use during early pregnancy

**DOI:** 10.1186/s12884-025-08190-y

**Published:** 2025-10-08

**Authors:** Kelly C. Young-Wolff, Felicia W. Chi, Cynthia I. Campbell, Monique B. Does, Christina N. Wysota, Deborah Ansley, Carley Castellanos, Gwen T. Lapham

**Affiliations:** 1 Division of Research, Kaiser Permanente Northern California, 4480 Hacienda Drive, Building B, Pleasanton, CA 94588 USA; 2 Department of Psychiatry and Behavioral Sciences, University of California, San Francisco, CA USA; 3 Department of Population Health, New York University, New York, NY USA; 4 Regional Offices, Kaiser Permanente Northern California, Pleasanton, CA USA; 5 Health Research Institute, Kaiser Permanente Washington, Seattle, WA USA; 6 Department of Health Systems and Population Health, University of Washington, Seattle, WA USA

**Keywords:** Cannabis, Marijuana, Pregnancy, Preconception, Frequency

## Abstract

**Background:**

Cannabis use during pregnancy is increasingly common and is associated with adverse health outcomes for pregnant individuals and their offspring. Identifying preconception factors that are associated with prenatal cannabis use is critical to inform early prevention and intervention. This study tested whether frequency of preconception cannabis use was associated with cannabis use during early pregnancy using data from a large healthcare system with universal screening for cannabis use.

**Methods:**

This observational study included pregnant individuals in Kaiser Permanente Northern California (KPNC) who self-reported any cannabis use (daily, weekly, or monthly or less) during the year before pregnancy from 2011 to 2022 (excluding 2020). Prenatal cannabis use was based on self-report and/or a positive toxicology test during the first trimester of pregnancy at entrance to prenatal care (at ~ 8 weeks gestation). Modified Poisson models were fit to assess associations between frequency of preconception cannabis use and prenatal cannabis use, adjusting for covariates.

**Results:**

The sample of 40,806 pregnancies from 36,622 unique individuals who self-reported any preconception cannabis use was 65.7% non-White; 27.6% were aged < 25 years. Nearly half (45.1%) screened positive for prenatal use, including 23.7% by self-report and 36.6% by positive toxicology test. Compared to monthly or less preconception cannabis use, daily use (adjusted prevalence ratio [aPR] = 2.66, 95% CI 2.59–2.73) or weekly use (aPR = 1.99, 95% CI 1.93–2.05) was associated with greater risk of prenatal cannabis use. Results were similar when prenatal cannabis use was based on self-report only or on toxicology testing only.

**Conclusions:**

Greater frequency of preconception cannabis use was associated with substantially increased risk of prenatal cannabis use. Findings reinforce the need for early harm prevention efforts focused on reducing the frequency of cannabis use among women of reproductive age, including screening, education, and early linkage to intervention.

**Supplementary Information:**

The online version contains supplementary material available at 10.1186/s12884-025-08190-y.

## Introduction

Cannabis use and cannabis use disorder are becoming increasingly prevalent among pregnant women in the US [1, 2]. Prenatal cannabis use is associated with significant health risks for pregnant women and their offspring, including preeclampsia, gestational hypertension, placental abruption, preterm birth, small-for-gestational-age infant, and admission to the neonatal intensive care unit [3–10]. As the strength of cannabis products increases in the US [11], there are growing concerns about the health risks of prenatal cannabis use, and national medical organizations recommend abstinence from cannabis during pregnancy [10, 12].

Understanding preconception factors that are associated with greater risk of using cannabis during pregnancy is critically important, as this knowledge could inform targeted prevention and intervention efforts before conception. Pregnant women often quit or cut down on their cannabis use over the course of pregnancy [13, 14], and initial studies with smaller convenience samples suggest that women who use cannabis more frequently prior to pregnancy recognition are more likely to continue to use after pregnancy recognition [15]. While more frequent cannabis use before pregnancy may also be a risk factor for prenatal cannabis use, especially before pregnancy recognition [16], this relationship is understudied. If more frequent preconception cannabis use is strongly associated with prenatal use, efforts to implement screening for frequency of cannabis for reproductive aged women in women’s health settings more broadly may be warranted.

Using population-based data from a large health care system with universal screening for cannabis use both before and during pregnancy, we tested whether more frequent preconception cannabis use was associated with cannabis use during early pregnancy.

## Methods

This cross-sectional observational study was conducted in Kaiser Permanente Northern California (KPNC) which universally screens for preconception and prenatal cannabis use at entry to prenatal care (at ~ 8 weeks gestation) through self-report and a urine toxicology test. The sample included pregnant individuals screened from 2011 to 2022 (excluding 2020 due to an electronic health record [EHR] system change that temporarily comprised data on self-reported prenatal substance use) who self-reported any cannabis use during the year before pregnancy (eFigure). This study followed STROBE guidelines and was approved by the KPNC IRB with a waiver of informed consent.

Preconception cannabis use was based on the frequency of self-reported cannabis use during the year before pregnancy, and prenatal cannabis use was based on any self-reported use since pregnancy at entry to prenatal care or a positive toxicology test during the first trimester (prenatal) (eMethods). Covariates were based on prior literature and availability in the electronic health record (EHR), including sociodemographic characteristics (age, race and ethnicity, neighborhood deprivation, Medicaid insurance coverage), parity, body mass index, and medical conditions diagnosed during the year before pregnancy onset, including psychiatric disorders (anxiety, depressive disorder and other), substance use disorders (cannabis use disorder, tobacco use disorder, and other alcohol and drug use disorders), sleep problems, common pain conditions (eMethods) and year of screen. Modified Poisson models with robust error variance were fit to assess associations between frequency of preconception cannabis use and prenatal cannabis use, adjusting for covariates and accounting for correlated observations of individuals with more than one pregnancy during the study period. In addition, we conducted two sets of sensitivity analyses. First, we refit the model excluding individuals who received a toxicology test before 30 days of gestation to minimize detection of cannabis use that occurred only during preconception, as heavy cannabis use may be detectable for up to 30 days. Second, we fit models with and without adjusting for cannabis use disorder to assess the impact of potential collinearity. Two-sided P values < 0.05 were considered statistically significant.

## Results

Among 40,806 pregnancies from 36,622 unique individuals who self-reported any preconception cannabis use (23.4% daily, 21.0% weekly, 55.6% monthly or less), 65.7% were non-White and 27.6% were aged < 25 years. The self-reported prenatal cannabis questionnaire and toxicology testing occurred at a median (IQR) of 51.0 (40.0–61.0) days gestation and 58.0 (51.0–71.0) days gestation, respectively. Nearly half (45.1%) screened positive for prenatal use, including 23.7% by self-report and 36.6% by toxicology testing (Table 1). Greater frequency of preconception cannabis use was associated with younger age, non-White race, Medicaid insurance, obesity, and psychiatric, substance use and common pain conditions.

**Table 1:** Patient characteristics among pregnant individuals who self-reported preconception cannabis use, 2011–2019, 2021–2022 (*N* = 40,806)

			Frequency of preconception cannabis use:					
	All		Monthly (*N* = 22,683, 55.6%)	Weekly (*N* = 8,570, 21.0%)			Daily (*N* = 9,553, 23.4%)	
	N	%	N	%	N	%	N	%
Age group								
14–17	757	1.9	356	1.6	189	2.2	212	2.2
18–24	10,472	25.7	4,691	20.7	2,333	27.2	3,448	36.1
25–34	22,876	56.1	13,355	58.9	4,659	54.4	4,862	50.9
35+	6,701	16.4	4,281	18.9	1,389	16.2	1,031	10.8
Race and ethnicity								
Asian	4,099	10.1	2,884	12.7	662	7.7	553	5.8
Black	5,723	14.0	2,446	10.8	1,239	14.5	2,038	21.3
Hispanic	10,775	26.4	5,861	25.8	2,213	25.8	2,701	28.3
White	18,063	44.3	10,347	45.6	3,995	46.6	3,721	39.0
Other/Unknown	2,146	5.3	1,145	5.1	461	5.4	540	5.7
NDI quartile								
1st	6,923	17.0	4,082	18.0	1,435	16.7	1,406	14.7
2nd	7,654	18.8	4,323	19.1	1,587	18.5	1,744	18.3
3rd	10,991	26.9	6,180	27.3	2,299	26.8	2,512	26.3
4th	15,238	37.3	8,098	35.7	3,249	37.9	3,891	40.7
Parity								
0	23,805	58.3	13,311	58.7	4,967	58.0	5,527	57.9
1	9,583	23.5	5,450	24.0	2,048	23.9	2,085	21.8
2+	4,654	11.4	2,530	11.2	962	11.2	1,162	12.2
Unknown	2,764	6.8	1,392	6.1	593	6.9	779	8.2
Body mass index								
Underweight	822	2.0	369	1.6	173	2.0	280	2.9
Normal	16,370	40.1	9,420	41.5	3,432	40.1	3,518	36.8
Overweight	11,222	27.5	6,357	28.0	2,331	27.2	2,534	26.5
Obese	12,223	30.0	6,440	28.4	2,593	30.3	3,190	33.4
Unknown	169	0.4	97	0.4	41	0.5	31	0.3
Medicaid	6,112	15.0	2,614	11.5	1,378	16.1	2,120	22.2
Psychiatric diagnosis								
Anxiety or depressive disorder	8480	20.8	4,265	18.8	1,877	21.9	2,338	24.5
Other psychiatric disorders	2,195	5.4	968	4.3	522	6.1	705	7.4
SUD diagnosis								
Cannabis use disorder	1,072	2.6	276	1.2	257	3.0	539	5.6
Tobacco use disorder	1,339	3.3	554	2.4	311	3.6	474	5.0
Other alcohol or drug use disorders	903	2.2	379	1.7	222	2.6	302	3.2
Sleep problems	997	2.4	552	2.4	218	2.5	227	2.4
Common pain conditions	18,090	44.3	9,647	42.5	3,810	44.5	4,633	48.5
Prenatal cannabis use								
Self-report or urine toxicology test	18,387	45.1	6001	26.5	4784	55.8	7602	79.6
Self-report	9,673	23.7	2855	12.6	2559	29.9	4259	44.6
Urine toxicology test	14,953	36.6	4110	18.1	3853	45.0	6990	73.2

Cannabis use in the year before pregnancy (preconception) was based on self-report, and cannabis use in the first trimester of pregnancy (prenatal) was based on positive self-report or positive urine toxicology test; all toxicology tests were confirmed with a confirmatory laboratory test. First trimester is defined as 90 days from last menstrual period. The 1 st NDI quartile indicates the lowest NDI (highest socioeconomic status), and the 4th quartile indicating the highest NDI (lowest socioeconomic status). Other psychiatric diagnoses include attention-deficit/hyperactivity disorder, bipolar disorder, personality disorder, post-traumatic stress disorder and psychotic disorder

*NDI * Neighborhood deprivation index, *SUD * Substance use disorder

**Fig. 1:**
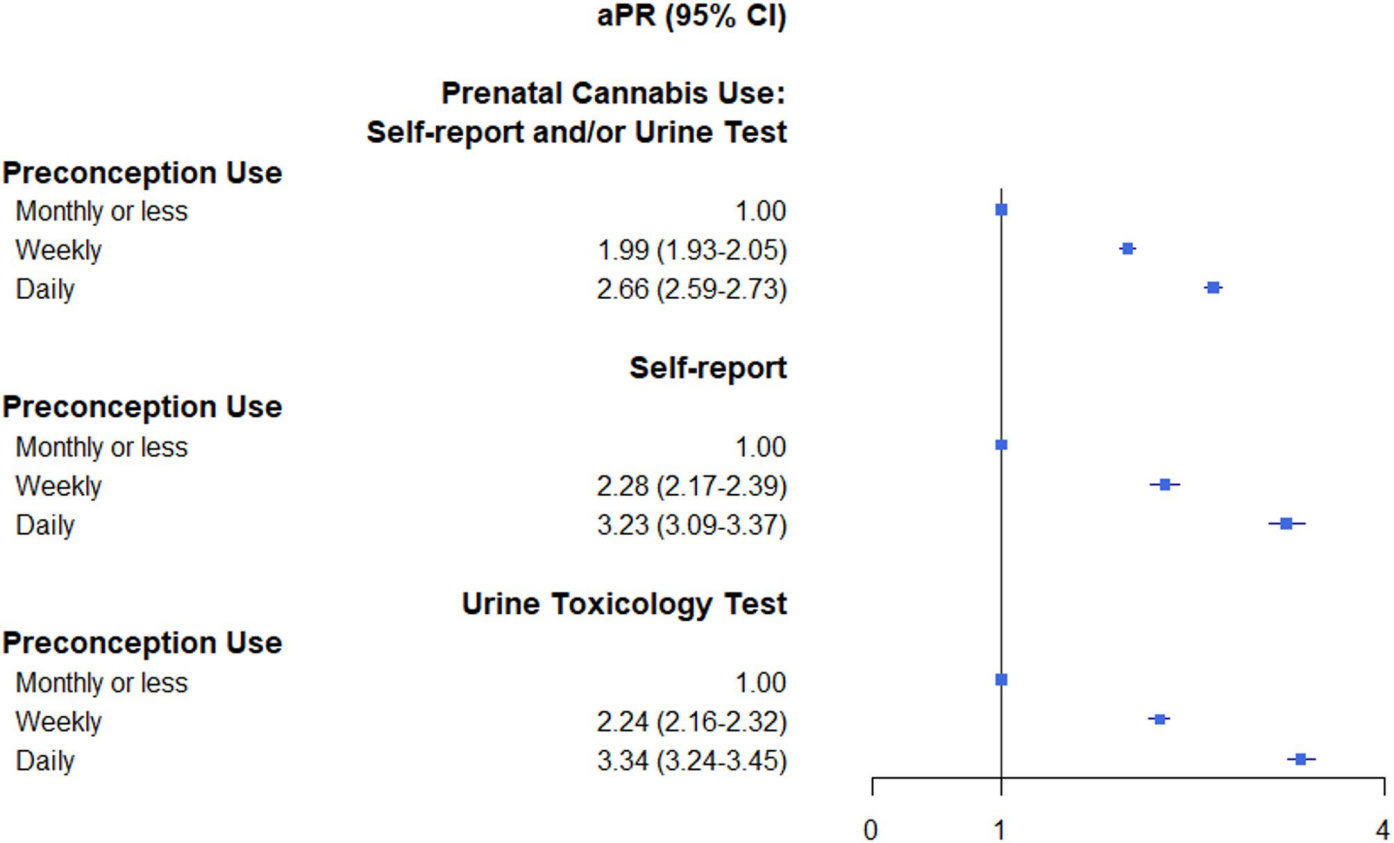
Associations between frequency of preconception cannabis use and prenatal cannabis use, among preconception cannabis users. Adjusted prevalence ratios (aPR) and 95% confidence intervals (95% CI) estimated from modified Poisson models with an exchangeable correlation matrix, comparing “weekly” and “daily” preconception use versus “monthly or less” preconception use, for outcome = any prenatal use based on self-report and/or positive urine toxicology test, self-report only, or positive urine toxicology test only. All models adjusted for age (14-17, 18-24, 25-34, ≥35), race and ethnicity (Asian, Black, Hispanic, White, Other), neighborhood deprivation index quartiles, parity (0, 1, ≥2, missing), body mass index (<18.5, 18.5-24.9, 25.0-29.9, ≥30.0, unknown), psychiatric disorders, substance use disorders, sleep problems, common pan conditions and year while accounting for multiple pregnancies per individual

Adjusted models found a dose-response relationship between frequency of preconception use and odds of prenatal cannabis use. Compared to individuals who reported monthly or less preconception cannabis use, the risk of any prenatal cannabis use were greater among those who self-reported daily (adjusted prevalence ratio [aPR] = 2.66, 95% CI 2.59–2.73) or weekly preconception use (aPR = 1.99, 95% CI 1.93–2.05) (Fig. 1). Similar results were observed when prenatal cannabis use by self-report (aPR = 3.23, 95% CI 3.09–3.37 for daily and aPR = 2.28, 95% CI 2.17–2.39 for weekly vs. monthly or less, respectively) and toxicology tests (aPR = 3.34, 95% CI 3.24–3.45 for daily and aPR = 2.24, 95% CI 2.16–2.32 for weekly vs. monthly or less) were examined separately. Results from sensitivity analyses that excluded those whose toxicology tests were conducted prior to 30 days gestation (~ 1%) found similar results, with greater odds of prenatal cannabis use via toxicology testing among those who self-reported daily (aPR = 3.35, 95% CI 3.24–3.46) or weekly preconception use (aPR = 2.24, 95% CI 2.16–2.32) (eTable 1). In addition, results from models with and without adjusting for cannabis use disorder were almost identical, suggesting minimal concern of collinearity (eTable 2).

## Discussion

Given the increasing prevalence of cannabis use among pregnant and reproductive-aged women and the expanding legalization of cannabis across the US, it is critical to understand preconception factors associated with prenatal cannabis use to inform early prevention and intervention. In this large study of > 40,000 pregnancies with self-reported preconception cannabis use, nearly half continued use during early pregnancy, with 80% of individuals who reported daily preconception use continuing to use into early pregnancy. These estimates of continued use during pregnancy are higher than those found by other studies [17, 18], likely because other research has typically examined cannabis use after pregnancy recognition and relied solely on self-reported prenatal cannabis use, which can underestimate the true prevalence [19].

There was a clear dose-response relationship between frequency of preconception cannabis use and greater odds of prenatal cannabis use, with those who used daily before pregnancy having about three times or higher risk of cannabis use during early pregnancy depending on prenatal cannabis screening method. Importantly, while results were strongest for prenatal cannabis use based on urine toxicology testing, findings were consistent across all measurement approaches (self-report, urine toxicology testing, or either). All models adjusted for key sociodemographic and behavioral covariates, highlighting preconception cannabis use as an independently important potential risk factor for prenatal cannabis use.

Our screening for prenatal cannabis use did not differentiate whether prenatal use only occurred before pregnancy recognition versus continued after. Women who use cannabis daily prior to conception may have greater odds of cannabis use during early pregnancy due to ongoing use prior to recognition of pregnancy, potentially due to the challenges of reducing or stopping frequent use [15, 20]. While quitting cannabis use at any point during pregnancy is beneficial, even cannabis use that is limited only to early pregnancy, particularly during critical periods of fetal development, may carry risks [21]. More frequent preconception cannabis use could also indicate more severe addiction and vulnerability to cannabis use even after pregnancy recognition, especially for those who rely on cannabis to manage mental health and medical symptoms [22–26]. Women with more frequent preconception cannabis use are also more likely to use multiple modes of cannabis administration (e.g., smoking, edibles, vaping) [27], which could further influence the risk of continued use during pregnancy. Future research is needed to better understand how different aspects of preconception cannabis use, including mode, product strength, and reasons for use, influence risk and duration of prenatal cannabis use.

Research indicates that most pregnant women who use cannabis also used it before pregnancy [28], and our results demonstrate that women who use more frequently (i.e., weekly, daily) during the preconception period are far more likely to continue cannabis use into the prenatal period. These findings point to the value of preconception screening for identifying women who may benefit from prevention and cessation efforts even prior to pregnancy [29]. However, despite recommendations for routine cannabis screening for adults in medical settings [30] and the availability of valid, brief screens for the frequency of cannabis use [31], preconception cannabis screening is not common. Efforts to integrate screening and clinician education to better support women during the preconception period are essential to reducing prenatal cannabis use [29].

Notably, those using cannabis on a daily or near daily basis may have a cannabis use disorder. Screening for preconception use frequency provides an opportunity for clinicians who recognize a possible use disorder to refer patients for further evaluation in specialty addiction medicine treatment. Some patients will have a harder time with the withdrawal symptoms and nausea that is often experienced after stopping cannabis because of the coexisting nausea and vomiting of early pregnancy, therefore making ongoing cessation efforts less successful. While treatment in pregnancy is encouraged, treatment prior to pregnancy is ideal for harm prevention for both mother and child.

Finally, our study indicated that those with younger age, non-White race, Medicaid insurance, obesity, and psychiatric, substance use and common pain conditions had a greater frequency of preconception cannabis use. Public health messaging and education strategies that prioritize these at-risk groups may be most beneficial.

### Strengths and limitations

Our study has important strengths, including a large, diverse sample of pregnant individuals universally screened for preconception and prenatal cannabis use. Our measure of prenatal cannabis use was based on self-report and urine toxicology testing, minimizing misclassification of our outcome. All analyses adjusted for key sociodemographic and health-related covariates available in the EHR, increasing the validity of our findings. The sample was large enough to examine differences by type of cannabis screening.

Our study also has several limitations. The sample comprised pregnant individuals in a large healthcare system in Northern California who screened positive for preconception cannabis use during standard prenatal care, potentially limiting generalizability to uninsured individuals, those who do not come in for prenatal care, or those outside of California. Preconception cannabis use was based on self-report at the time of prenatal screening which could underestimate use due to stigma or recall bias, and findings may not generalize to individuals who choose not to disclose their use. Further, we did not have information on preconception cannabis product strength or mode of administration. Future studies should examine these factors to better understand risk factors for cannabis use during pregnancy.

While our study uniquely assessed any cannabis use during early pregnancy regardless of pregnancy recognition, urine toxicology tests may have detected residual frequent preconception cannabis use. However, this is unlikely to explain the observed associations given that urine toxicology tests typically detect cannabis use for up to 30 days in heavy users, and the median gestational age of urine toxicology testing was 58.0 days (IQR: 51.0–71.0). Further, sensitivity analyses that excluded those with toxicology tests before 30 days gestation found comparable results, suggesting that results are not driven by misclassification of prenatal cannabis use. Finally, this study was conducted among pregnant individuals with preconception cannabis use, and the sample size was not large enough to fit multivariable models examining frequency of prenatal cannabis use as the outcome.

## Conclusions

In this large study of pregnant women with preconception cannabis use, nearly half screened positive for cannabis use during early pregnancy. Higher frequency of preconception use was strongly associated with increased odds of prenatal cannabis use. Findings highlight the potential importance of screening for and addressing frequent cannabis use among women of reproductive age even prior to pregnancy. Routine screening, patient resources, and early referrals during early pregnancy may help to reduce prenatal cannabis use and improve maternal and child health. Additional research is needed to examine how specific aspects of preconception cannabis use, include mode of administration, product strength, and reasons for use, influence risk of prenatal cannabis use to inform more tailored interventions.

## Supplementary Information

Supplementary Material 1

## Data Availability

The datasets generated and/or analysed during the current study are not publicly available due to KPNC IRB regulation but are available from the corresponding author on reasonable request.
