# Supplementary material for: Association of preconception cannabis use frequency with cannabis use during early pregnancy
Source: BMC Pregnancy Childbirth. 2025 Oct 8;25:1044. doi: 10.1186/s12884-025-08190-y (PMC12506406; doi:10.1186/s12884-025-08190-y)
Supplement: Supplementary file 1 — Supplementary Material 1 [file 12884_2025_8190_MOESM1_ESM.docx]

**Supplementary Online Content**

eFigure. STROBE Diagram of Patient Selection

eMethods.

1. Measure of Cannabis Use Frequency
2. ICD-9-CM and ICD-10-CM Diagnosis Codes Used to Identify Psychiatric Disorders, Substance Use Disorders, Sleep Problems and Common Pain Conditions
3. Analysis

eTables. Sensitivity Analysis Results

1. Associations between frequency of preconception cannabis use and prenatal cannabis use, among the sample with urine toxicology test occurring at least 30 days into the pregnancy
2. Associations between frequency of preconception cannabis use and prenatal cannabis use, comparing models with and without adjusting for cannabis use disorder

**eFigure. STROBE Diagram of Patient Selection**

***Pregnant individuals with*** ***outcome dates between Jan 1, 2011 to December 31, 2022***

n of pregnancies = 721,464; n of individuals = 447,813

***Final analytical sample***

n of pregnancy = 40,806; n of individuals = 36,622

Excluded those with no preconception cannabis use (n = 321,758, 88.7%)

*Notes.* KPNC = Kaiser Permanente Northern California. PSQ = Prenatal Screening Questionnaire. UDAP = Urine Toxicology Test. *Data for year 2020 were not included due to changes in care during the early COVID-19 pandemic and an EHR system change in early 2020 that temporarily compromised self-reported prenatal substance use data capture.

Excluded those with missing data on neighborhood deprivation index

(n = 179, 0.05%)

***With valid urine toxicology data on cannabis use in 1^st^ trimester***

n of pregnancy = 362,743; n of individuals = 275,921

Excluded those who did not have valid UDAP data on cannabis use in 1^st^ trimester (n = 46,526, 11.4%)

***With valid data on cannabis use 1 year prior to pregnancy and in 1^st^ trimester***

n of pregnancy = 409,269; n of individuals = 300,439

Excluded those who did not have valid data on cannabis use:

1. 1 year prior to pregnancy (n=2,715, 0.6%);
2. since pregnancy (n=2,614, 0.6%)
3. those whose PSQ was conducted in year 2020* (n=43,218, 9.4%)

***Enrolled as KPNC member during pregnancy*** who ***had prenatal care office visit*** and ***PSQ screening in 1^st^ trimester***

n of pregnancy = 457,816; n of individuals= 326,094

Excluded those who:

1. did not have KP membership during pregnancy (n=18,281, 2.5%);
2. did not have any prenatal office visit during pregnancy (n=119,657, 16.6%);
3. did not have valid PSQ screening in 1^st^ trimester (n=125,710, 17.4%)

**eMethods.**

1. **Measure of Cannabis Use Frequency**

Frequency of preconception cannabis use during the year before pregnancy was measured via a single item on the prenatal screening questionnaire (i.e., In the 12 months before you were pregnancy, how often have you used marijuana? [never, monthly or less, weekly, daily]). Frequency of prenatal cannabis use during the first trimester was based on 1) self-reported frequency measured via a single item on the prenatal screening questionnaire (i.e., Since pregnancy, how often have you used marijuana? [never, monthly or less, weekly, daily]) that were conducted within 90 days from last menstrual period and 2) urine toxicology test results (within 90 days from last menstrual period).

1. **ICD-9-CM and ICD-10-CM Diagnosis Codes Used to Identify Psychiatric Disorders, Substance Use Disorders, Sleep Problems and Common Pain Conditions**
2. Psychiatric Disorders

Anxiety disorder

ICD-9-CM: 293.84, 300.0, 300.00, 300.01, 300.02, 300.09, 300.20, 300.21, 300.22, 300.23, 300.29, 300.3, 309.21, 309.24, 309.81, 309.89

ICD-10-CM: F06.4, F40.00, F40.01, F40.02, F40.9, F40.10, F40.11, F40.210, F40.218, F40.220, F40.228, F40.230, F40.231, F40.232, F40.233, F40.240, F40.241, F40.242, F40.243, F40.248, F40.290, F40.291, F40.298, F40.8, F41, F41.0, F41.1, F41.3, F41.8, F41.9, F42, F42.2, F42.3, F42.4, F42.8, F42.9, F43.22, F43.8

Attention-deficit/hyperactivity disorder (ADHD)

ICD-9-CM: 314.00, 314.01, 314.1, 314.2, 314.8, 314.9

ICD-10-CM: F90.0, F90.1, F90.2, F90.8, F90.9

Bipolar disorder

ICD-9-CM: 296.0*, 296.1*, 296.4*, 296.5*, 296.6*, 296.7, 296.80, 296.81, 296.89, 301.13

ICD-10-CM: F30*, F31*, F34.0

Depressive disorder

ICD-9-CM: 296.2*, 296.3*, 296.82, 296.90, 296.99, 300.4, 301.12, 309.0, 309.1, 309.28, 311, 648.40, 648.41, 648.42, 648.43, 648.44

ICD-10-CM: F32*, F33*, F34.1, F34.8, F34.89, F34.9, F39, F43.21, F43.23, O99.34

Personality disorder

ICD-9-CM: 301.0, 301.10, 301.11, 301.12, 301.13, 301.20, 301.21, 301.22, 301.3, 301.4, 301.50, 301.51, 301.59, 301.6, 301.7, 301.81, 301.82, 301.83, 301.84, 301.89, 301.9

ICD-10-CM: F60.0, F60.1, F60.2, F60.3, F60.4, F60.5, F60.6, F60.7, F60.81, F60.89, F60.9, F68.12

Posttraumatic stress disorder (PTSD)

ICD-9-CM: 309.81

ICD-10-CM: F43.10, F43.11, F43.12

Psychotic disorder

ICD-9-CM: 295*, 297*, 298.0, 298.1-298.9

ICD-10-CM: F20, F25, F22, F23, F28, F29

1. Substance Use Disorders

Cannabis use disorder

ICD-9-CM: 304.3*, 305.2*

ICD-10-CM: F12*

Tobacco use disorder

ICD-9-CM: 305.1*

ICD-10-CM: F17*, Z71.6

Other alcohol and drug use disorders

ICD-9-CM: 291*, 303*, 305.0*

ICD-10-CM: F10.1*, F10.2*, F10.9*

1. Sleep Problems

ICD-9-CM: 307.4*, 327, 327.01, 327.02, 327.09, 327.1*, 327.2*, 327.3*, 327.4*, 327.5*, 327.8

ICD-10-CM: A81.83, F11.182, F13.982, F14.182, F15.282, F19.982, F51.0*, F51.1*, F51.3, F51.4, F51.5, F51.8, F51.9, G47, G47.0*, G47.1*, G47.2*, G47.3*, G47.5*, G47.6*, G47.8, G47.9, R06.83, Z72.82, Z72.820, Z72.821

1. Common Pain Conditions

Common pain conditions were defined based on the list of ICD-9-CM and ICD-10-CM diagnosis codes previously published.

Mayhew M, DeBar LL, Deyo RA, Kerns RD, Goulet JL, Brandt CA, Von Korff M. Development and Assessment of a Crosswalk Between ICD-9-CM and ICD-10-CM to Identify Patients with Common Pain Conditions. J Pain. 2019 Dec;20(12):1429-1445. doi: 10.1016/j.jpain.2019.05.006. Epub 2019 May 23. PMID: 31129316; PMCID: PMC6874733.

Lapham G, Boudreau DM, Johnson EA, Bobb JF, Matthews AG, McCormack J, Liu D, Samet JH, Saxon AJ, Campbell CI, Glass JE, Rossom RC, Murphy MT, Binswanger IA, Yarborough BJH, Bradley KA; PROUD Collaborative Investigators. Prevalence and treatment of opioid use disorders among primary care patients in six health systems. Drug Alcohol Depend. 2020 Feb 1;207:107732. doi: 10.1016/j.drugalcdep.2019.107732. Epub 2019 Nov 15. PMID: 31835068; PMCID: PMC7158756

1. **Analysis**

To estimate the associations between frequency of preconception cannabis use and prenatal cannabis use, we fit modified Poisson models^1^ with an exchangeable correlation matrix to account for nonindependence of multiple pregnancies from some individuals, adjusting for socio-demographic and clinical characteristics. Adjusted prevalence ratios (aPR) and 95% confidence intervals (95% CI) comparing “weekly” and “daily” preconception use versus “monthly or less” preconception use were estimated from the models adjusting for age (14-17, 18-24, 25-34, ≥35), race and ethnicity (Asian, Black, Hispanic, White, Other), neighborhood deprivation index quartiles, parity (0, 1, ≥2, missing), body mass index (<18.5, 18.5-24.9, 25.0-29.9, ≥30.0, unknown), psychiatric disorders, substance use disorders, sleep problem, common pan conditions and year while accounting for multiple pregnancies per individual. We conducted all analyses using SAS 9.4.

We also conducted two sets of sensitivity analyses. First, we refit the model to examine associations between frequency of preconception cannabis use and prenatal cannabis use based on urine toxicology test among the sample with urine toxicology test occurring at least 30 days into the pregnancy, in order to minimize detection of cannabis use that occurred only during preconception, as heavy cannabis use may be detectable for up to 30 days. Second, we fit models with and without adjusting for cannabis use disorder to assess the impact of potential collinearity.

1. Zou GY, Donner A. Extension of the modified Poisson regression model to prospective studies with correlated binary data. Stat Methods Med Res. Dec 2013;22(6):661-70. doi:10.1177/0962280211427759

**eTables. Sensitivity Analysis Results**

eTable 1. Associations between frequency of preconception cannabis use and prenatal cannabis use, among the sample with urine toxicology test occurring at least 30 days into the pregnancy

|  | Prenatal use based on  urine toxicology test | |
| --- | --- | --- |
|  | aPR, 95% CI | P value |
| **Frequency of preconception cannabis use** |  |  |
| Weekly vs Monthly | **2.24 (2.16, 2.32)** | **<.001** |
| Daily vs Monthly | **3.35 (3.24, 3.46)** | **<.001** |

*Notes*. Adjusted prevalence ratios (aPR) and 95% confidence intervals (95% CI) estimated from a modified Poisson model with an exchangeable correlation matrix, comparing “weekly” and “daily” preconception use versus “monthly or less” preconception use, for outcome = any prenatal use based on positive urine toxicology test, among the sample with urine toxicology test occurring at least 30 days into the pregnancy. All models adjusted for age (14-17, 18-24, 25-34, ≥35), race and ethnicity (Asian, Black, Hispanic, White, Other), neighborhood deprivation index quartiles, parity (0, 1, ≥2, missing), body mass index (<18.5, 18.5-24.9, 25.0-29.9, ≥30.0, unknown), psychiatric disorders, substance use disorders, sleep problems, common pan conditions and year while accounting for multiple pregnancies per individual.

eTable 2. Associations between frequency of preconception cannabis use and prenatal cannabis use, comparing models with and without adjusting for cannabis use disorder

|  | Model including CUD |  | Model not including CUD |
| --- | --- | --- | --- |
|  | aPR (95% CI) |  | aPR (95% CI) |
| **Outcome = prenatal MAR use, self-report and/or urine test** | | | |
| Preconception use: |  |  |  |
| Monthly or less | 1.00 |  | 1.00 |
| Weekly | 1.99 (1.93, 2.05) |  | 1.99 (1.94, 2.05) |
| Daily | 2.66 (2.59, 2.73) |  | 2.67 (2.60, 2.73) |
|  |  |  |  |
| **Outcome = prenatal MAR use, self-report** | | | |
| Preconception use: |  |  |  |
| Monthly or less | 1.00 |  | 1.00 |
| Weekly | 2.28 (2.17, 2.39) |  | 2.28 (2.17, 2.39) |
| Daily | 3.23 (3.09, 3.37) |  | 3.24 (3.10, 3.38) |
|  |  |  |  |
| **Outcome = prenatal MAR use, urine test** | | | |
| Preconception use: |  |  |  |
| Monthly or less | 1.00 |  | 1.00 |
| Weekly | 2.24 (2.16, 2.32) |  | 2.24 (2.16, 2.32) |
| Daily | 3.34 (3.24, 3.45) |  | 3.35 (3.25, 3.46) |

*Notes*. Adjusted prevalence ratios (aPR) and 95% confidence intervals (95% CI) estimated from a modified Poisson model with an exchangeable correlation matrix, comparing “weekly” and “daily” preconception use versus “monthly or less” preconception use, for outcome = any prenatal use based on self-report and/or urine test, self-report only or positive urine toxicology test only. All models adjusted for age (14-17, 18-24, 25-34, ≥35), race and ethnicity (Asian, Black, Hispanic, White, Other), neighborhood deprivation index quartiles, parity (0, 1, ≥2, missing), body mass index (<18.5, 18.5-24.9, 25.0-29.9, ≥30.0, unknown), psychiatric disorders, other substance use disorders, sleep problems, common pan conditions and year while accounting for multiple pregnancies per individual.
